# An Off-Label Use of a Unibody Aortic Stent-Graft System for the Treatment of Infrarenal Abdominal Aortic Dissections

**DOI:** 10.1155/2019/6853135

**Published:** 2019-04-08

**Authors:** Joseph Faraj, Rebekah L. W. Tan, Bibombe P. Mwipatayi

**Affiliations:** ^1^Department of Vascular Surgery, Royal Perth Hospital, Perth, Western Australia, Australia; ^2^Department of Vascular Surgery, Hollywood Private Hospital, Western Australia, Australia; ^3^School of Medicine, University of Western Australia, Perth, Western Australia, Australia; ^4^Perth Institute of Vascular Surgery, Perth, Western Australia, Australia

## Abstract

Infrarenal abdominal aortic dissections (IAAD) are exceedingly rare, accounting for 1-4% of all aortic dissections. The evidence is scarce on how to best manage IAAD when they become symptomatic. Two main interventional approaches exist, open surgery and the endovascular approach. Conventional stent-graft systems make it difficult to treat nonaneurysmal aortic disease due to limb competition in a narrow distal aorta. Thus, we present a novel use of the Endologix Anatomical Fixation 2 (AFX2) Abdominal Aortic Aneurysm (AAA) endograft system for the treatment of four patients with IAAD. We also highlight an individual case study that was treated with an alternative endovascular approach and the complications that followed. This was to highlight and compare our successful experience with Endologix AFX2 AAA endograft system. There were multiple benefits for choosing this stent-graft; however the main advantage is its suitability in the narrow distal aorta. Our aim was to highlight an alternative endovascular approach for the successful treatment of a rare, challenging, and potentially fatal pathology.

## 1. Introduction

Infrarenal abdominal aortic dissections (IAAD) are exceedingly rare with most aortic dissections originating in the ascending thoracic aorta. Infrarenal abdominal aortic dissections (IAADs) account for 1% to 4% of all aortic dissections [1–3]. They can be classified etiologically as iatrogenic, spontaneous, or traumatic [1, 2]. Males tend to be most commonly affected with a median age of 60 years, usually with concomitant hypertension [2]. Due to the rarity of (IAAD) and the literature comprising mostly of case series there is no established diagnostic criteria or standards for therapeutic management regarding optimal imaging modality and choice of surgical procedure [4]. Treatment options for IAADs include conservative medical management with surveillance, open surgery, and endovascular repair (EVAR) [1, 5, 6].

We report the clinical features and novel use of the Endologix AFX2 AAA endograft system for the treatment of four patients who presented to our institution with IAADs. Special emphasis is placed on the clinical presentation of this rare entity and the endovascular treatment and complications experienced within our institution.

## 2. Case 1

**Figure 1:**
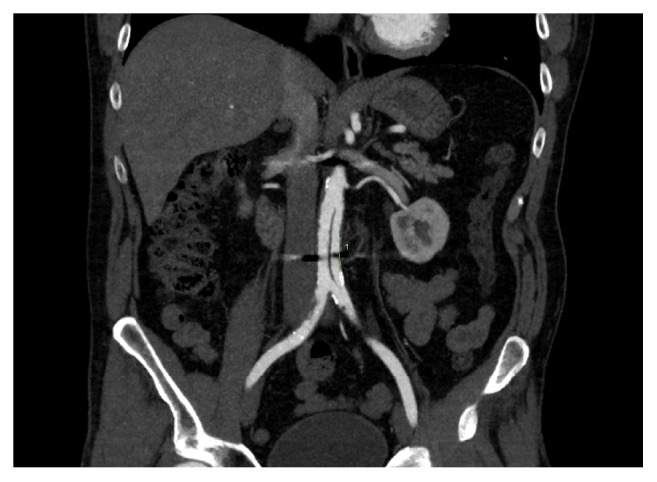
Computed-tomography angiography coronal slice demonstrating the infrarenal aortic dissection.

**Figure 2:**
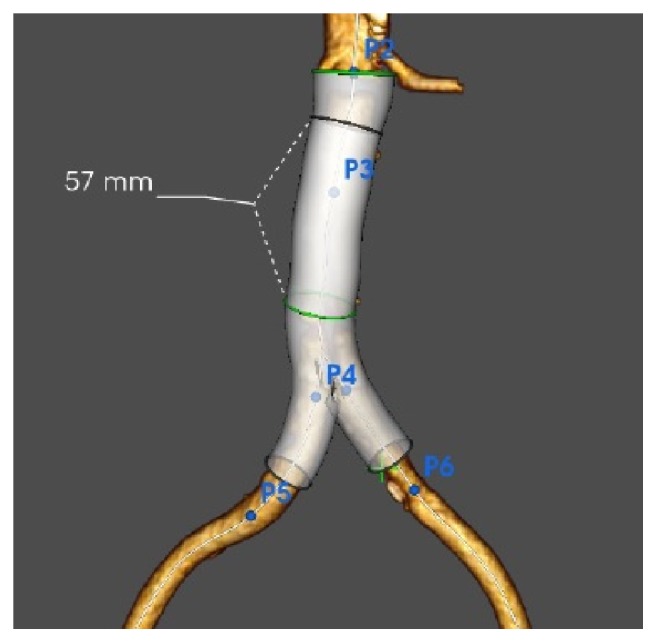
Treatment of the infrarenal aortic dissection using the Endologix AFX stent-graft system.

BH is a 59-year-old male presented after a high-speed motor vehicle accident. Screening Computed-Tomography (CT) imaging was carried out to exclude any injuries, revealing a L3 fracture and infrarenal aortic dissection. Dedicated CT angiography revealed a 7cm dissection in the infrarenal abdominal aorta extending into the proximal left common iliac artery (CIA) (Figure 1). The patient was initially managed conservatively with yearly surveillance over three years; however due to severe, uncontrolled hypertension the decision was made to treat. The patient was treated endovascularly using an AFX2 bifurcated AAA endograft sysytem (Endologix, Irvine, CA, USA) (Figure 2). The procedure went with no complications. At six-month follow-up the stent-graft was patent with no evidence of endoleak.

## 3. Case 2

**Figure 3:**
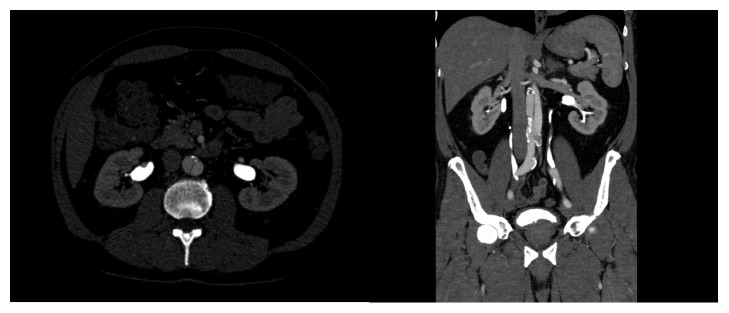
Computed-tomography angiography in cross-sectional and coronal view demonstrating the infrarenal aortic dissection.

A 73-year-old male presented with an incidental finding of an IAAD extending to the left CIA on CT angiography (Figure 3). Due to uncontrolled hypertension the patient was treated endovascularly using the AFX2 bifurcated AAA endograft system. The procedure went without any complications. At 12-month follow-up the stent-graft was patent with no evidence of endoleak.

## 4. Case 3

The case is a 56-year-old male who underwent a CT angiogram as a work-up for prostate surgery. There is an incidental finding of a 3.2cm infrarenal abdominal aortic aneurysm (AAA) with dissection extending distally involving both common iliac arteries (Figure 3). Due to uncontrolled hypertension the patient was treated endovascularly using the Endologix AFX2 bifurcated AAA endograft system. The procedure went without any complications. At 12-month follow-up the stent-graft was patent with no evidence of endoleak.

## 5. Case 4

**Figure 4:**
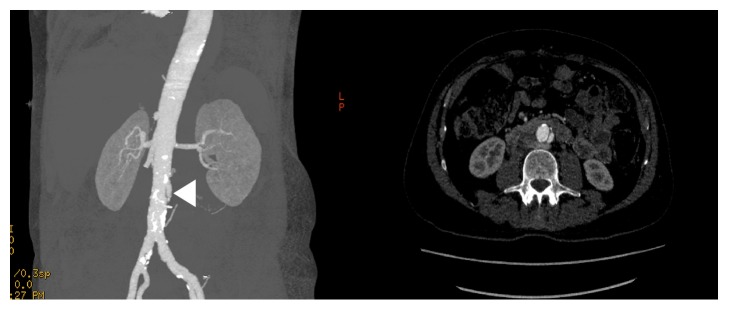
Computed-tomography angiography reformats demonstrating the infrarenal dissection on the cross-sectional plane and small saccular aneurysm in the coronal plane (arrowhead).

The case is a 70-year-old female with an infrarenal aortic dissection and an associated AAA which showed progression at surveillance. CT angiography showed a minor eccentric saccular aneurysm measuring 23mm involving the right anterolateral aspect, extending over a 9mm intimal flap (Figure 4). Due to uncontrolled hypertension and aneurysm being saccular in nature the patient was treated endovascularly using the Endologix AFX2 bifurcated AAA endograft system. The procedure went without any complications. At 12-month follow-up the stent-graft was patent with no evidence of endoleak.

## 6. Case 5

**Figure 5:**
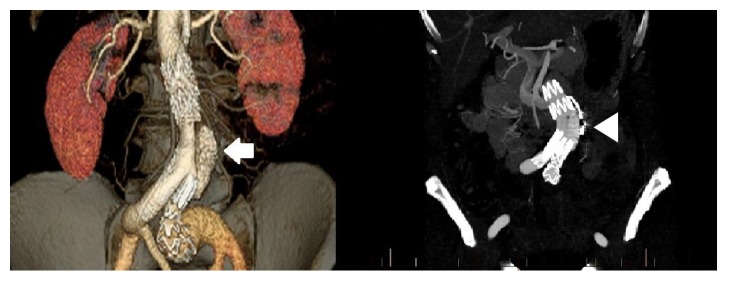
Computed-tomography angiography reconstruction formats demonstrating perfusion of the false lumen with mild aneurysmal dilatation distal to the infrarenal aortic stent.

A 71-year-old female presented with a pulsatile mass in her left groin causing significant discomfort. CT angiography showed a dissection involving the infrarenal abdominal aorta extending from the level of the inferior mesenteric artery into an aneurysmal left common iliac artery (CIA) measuring 29mm x 27mm. The patient was treated with a 24mm x 56mm Zenith® Spiral-Z® AAA Iliac Leg Graft (COOK medical, Bloomington, IN, USA) and deployed into the infrarenal aorta. Kissing iliac stents were deployed to exclude both the dissection at its distal point and the left CIA aneurysm. At 12-month follow-up CT angiography demonstrated exclusion of both the dissection and the left CIA aneurysm. However, CT angiography at 48-month follow-up demonstrated a type 2 endoleak with filling of the false lumen of the dissection in the infrarenal aorta with associated mild aneurysmal dilatation (Figure 5). The patient remained asymptomatic and no intervention was offered at that stage; however she remains under close routine surveillance.

## 7. Discussion

Infrarenal aortic dissections are rare. The increasing use of CT scanning for further investigation of undifferentiated abdominal pain has begun to reveal this condition more frequently [5]. The most common presenting symptoms are lower limb ischemia (33%), abdominal pain (30%), and back of flank pain (20%) [5]. Additional symptoms we observed in two of the six patients treated was erectile dysfunction. This presentation to our knowledge has not previously been reported in the literature.

The most common risk factors for spontaneous infrarenal aortic dissection are hypertension, hyperlipidaemia, and atherosclerosis and these factors were observed across all six patients. A dissection left untreated may result in the formation of an aneurysm that could ultimately rupture or lead to progressive stenosis leading to distal arterial insufficiency.

Nonpenetrating injuries of the abdominal aorta are rare, with only between 0.01% and 0.07% resulting in aortic dissection [7]. Despite this, more than 95% of aortic injuries are localised at the thoracic part; blunt abdominal aortic injuries are very rare and often associated with lesion of other intra-abdominal organs due to the protected anatomical position of the abdominal aorta [7]. Two major mechanisms have been postulated to explain this: direct from the blunt trauma with aortic compression against bony structures of the spine and indirectly because of the deceleration forces experienced in high-speed collisions [8]. Although most cases of blunt injury to the abdominal aorta present with no diagnostic problems, precise knowledge of the circumstances of trauma and a high suspicion index should lead to a CT scan or diagnostic angiography to rule this condition out [8, 9].

Therapeutic options in the case of chronic IAADs include close observation with regular follow-up, prosthetic replacement of the involved aorta, or open surgery [5]. Conservative treatment encompassing medical management and active surveillance is indicated in patients for patients with infrarenal aortic dissections. Endovascular interventions including angioplasty combined with stent-grafts, covered stents, or bare metal stents are indicated for patients with infrarenal abdominal aortic dissection which are symptomatic, large, or rapidly expanding or have morphological changes [1, 7]. All patients were treated endovascularly, with four out of the five patients treated using the Endologix AFX2 AAA endograft system (Endologix, Irvine, CA, USA) and one patient treated using a Zenith cook stent-graft with bilateral kissing iliac stents. The treatment of IAADs with the AFX2 AAA endograft system (Endologix, Irvine, CA, USA) is not within its instructions for use (IFU), as it is primarily designed for the treatment of AAAs. However, the same principle applies when treating IAAD, and this is to ensure active proximal fixation, accurate deployment, and exclusion of the pathology.

Endologix AFX2 AAA endograft system (Endologix, Irvine, CA, USA) differs from the other approved EVAR systems in several ways. It features the only unibody main body component, in contrast to the usual two- or three-component modular design. The device is deployed directly onto the aortic bifurcation, providing secure anatomic fixation, eliminating main body graft migration, and essentially relining the distal aorta and common iliac arteries. The base component is then complemented by an aortic extension cuff that is available in either an infrarenal or suprarenal option that provides seal through radial force and the ability of the compliant expanded polytetrafluoroethylene (ePTFE) graft material to accommodate irregularities in the aortic wall [10].

The prevalence of a narrow distal aorta (< 20 mm) associated with AAA may be as high as 65%; this was noted amongst our cohort with an average diameter of 17.8mm (range = 9.4mm – 19.8mm). In these situations, relining the aortic bifurcation with the Endologix AFX2 AAA endograft sysytem is more appealing than trying to squeeze two limbs side-by-side within a narrow lumen. Relining the aorta provides the ability to more aggressively postdilate the bifurcation to ensure an adequate-flow lumen and lower limb occlusion rates [10].

Finally, the main body device and aortic or iliac extensions are delivered through a single 17-Fr ID hydrophilic access sheath without the need for exchanges. The contralateral side is managed through a 7Fr sheath that is approved for standard percutaneous access and makes Endologix AFX2 AAA Endograft System the lowest profile system currently available [10]. The system can therefore be used in patients with small, diseased iliac access vessels without the need for a conduit, especially in patients with severe asymmetric disease on one side that can be safely traversed without the need for a larger limb delivery sheath. This was particularly useful in those patients where the dissection extended into the iliac vessels. This low-profile design and single indwelling delivery sheath also make Endologix AFX2 AAA Endograft System particularly well suited for percutaneous delivery [10].

All patients treated with the Endologix AFX2 AAA endograft system had no complications perioperatively and had no evidence of endoleak on follow-up at 12 months. Our one patient treated with the Zenith® Spiral-Z® AAA Iliac Leg Graft (COOK medical, Bloomington, IN, USA) and kissing iliac covered stents which was deployed proximal to the origin of the dissection unfortunately developed a type 2 endoleak and persistent filling of the false lumen of the dissection with expansion of the luminal diameter. The reason for this treatment choice was that the Endologix AFX2 AAA endograft system was not available at our institution.

Isolated infrarenal aortic dissections are a rare condition affecting both the young and elderly population. In those where aetiology is spontaneous, patients are often elderly with significant comorbidity. The use of the Endologix AFX2 AAA endograft System has been shown to be a safe and low-risk alternative to open aortic surgery in these populations.
